# Prevalence of modifiable factors limiting treatment efficacy of poorly controlled asthma patients: EFIMERA observational study

**DOI:** 10.1038/s41533-020-00189-6

**Published:** 2020-07-31

**Authors:** Paula Ribó, Jesús Molina, Myriam Calle, Luis Maiz, Carlos Campo, Paula Rytilä, Vicente Plaza, Antonio Valero

**Affiliations:** 1 Allergology Section, Pulmonology and Allergy Department, Hospital Clínic de Barcelona, Barcelona, Spain; 2 Grup d’Inmunoal·lèrgia Respiratoria Clínica i Experimental (IRCE), Institut d’investigacions Biomèdiques Agustí Pi i Sunyer (IDIBAPS), Barcelona, Spain; 3 Centro de Investigación Biomédica en Red de Enfermedades Respiratorias (CIBERES), Instituto de Salud Carlos III, Madrid, Spain; 4 Family Medicine Department, Centro de Salud Francia, Fuenlabrada, Madrid, Spain; 5 Pulmonology Departament, Hospital Clínico San Carlos, Madrid, Spain; 6 Medicine Department, Facultad de Medicina, Universidad Complutense de Madrid, Madrid, Spain; 7 Instituto de Investigación Biomédica, Hospital Clínico San Carlos (IdISSC), Madrid, Spain; 8 Pulmonology Department, Hospital Ramón y Cajal, Madrid, Spain; 9 Orion Pharma, Madrid, Spain; 10 Orion Corporation Orion Pharma, Espoo, Finland; 11 Respiratory Medicine Department, Hospital de la Santa Creu i Sant Pau, Barcelona, Spain; 12 Institut d’Investigació Biomédica Sant Pau (IIB Sant Pau), Barcelona, Spain; 13 Medicine Department, Facultat de Medicina, Universitat Autònoma de Barcelona, Bellaterra, Spain

**Keywords:** Health care, Medical research

## Abstract

Several modifiable factors leading to poor asthma control have been described. We aimed to determine the proportion of patients with inadequate treatment, adherence to it, or critical mistakes with inhaler technique, and their impact on asthma control. We conducted a cross-sectional multicenter observational study including asthma patients referred from primary to specialist care for the first time. Data collected were adequate prescription according to guidelines, treatment adherence, and disease control. Of the 1682 patients (age 45 ± 17 years, 64.6% men), 35.9% showed inadequate prescription, 76.8% low adherence, and 17% critical mistakes with inhaler technique, with significantly less critical mistakes among Easyhaler users versus other dry powder inhaler users (10.3 versus 18.4%; *p* < 0.05). Factors related to bad asthma control were inadequate prescription (OR: 3.65), non-adherence to treatment (OR: 1.8), and inhaler misuse (OR: 3.03). A higher number of risk factors were associated with a higher probability of having badly controlled asthma.

## Introduction

Asthma is a common disease and it accounts for considerable healthcare costs and loss of work productivity^1^. Its prevalence is increasing worldwide: nearly 1000 people die from asthma every day according to a study published in 2015^2^. Latest data about asthma global prevalence estimate that it affects 334 million people globally^3^. However, its prevalence varies greatly between countries with the highest prevalence seen in developed ones^1^. High direct (medications, hospitalization, Emergency Department care, etc.) and indirect (school absenteeism, loss of work productivity, etc.) costs are especially linked to exacerbations^3^.

Currently, asthma exacerbations are one of the most frequent causes of Emergency Department^4^ visits emphasizing the poor control of asthma in affected patients. Many factors can trigger an exacerbation^5^: exposure to external factors, poor adherence to treatment, inadequate therapy, and poor inhaler technique are among the most important modifiable causes in the case of uncontrollable asthma^6^. Several studies have demonstrated poor treatment adherence in asthmatic patients: <50% in children and 30–70% in adults, depending on the country, age, sex, and ethnicity^7,8^. The reasons for this variation were limited comprehension or recognition (among patients and/or physicians), beliefs or side effect concerns, inhaler characteristics, and instructions for their use. Frequently, the key aspect of poor asthma control is that many patients, especially with mild to moderate asthma, are undertreated^6^.

Thus asthma control as a therapeutic goal, is far from being achieved^9^. Better control would improve not only cost effectiveness but also quality of life, reduce school absenteeism, and increase work productivity as well^3^.

The same situation is reflected in primary care management of asthma. Several studies about asthma in primary care seem to suggest that asthma control in clinical practice is suboptimal despite available therapies. During the past years, little apparent improvement in symptom control has been documented by European publications^10,11^.

The primary objectives of this study are to (1) determine the appropriateness of prescribed treatment as well as the presence of poor adherence and critical mistakes in the inhalation technique in patients referred from primary care to a specialist for the first time; and (2) to study the relationship between these factors and poor asthma control. This real-life study may allow us to explore whether asthma management in primary care has improved in terms of quality of prescription, inhaler technique, and patients’ adherence to treatment, 30 years after the implementation of asthma guidelines in Spain.

## Results

The number of patients enrolled in the study was 1682, of whom 64.6% were men. The mean age was 45 ± 17 years. The characteristics of the cohort are shown in Table 1.

**Table 1:** Baseline demographic and clinical characteristics.

Variable	Results	Number of patients evaluated
Age (years), mean ± SD (range)	45.2 ± 17.9 (18–90)	1681
Females, *n* (%)	592 (35.4)	1670
Males, *n* (%)	1078 (64.6)	1670
Age at diagnosis (years), mean ± SD	33.8 ± 15.7	1682
Disease duration (years), mean ± SD	14.9 ± 14.1	1682
Time between treatment initiation and diagnosis (years), mean ± SD	1.1 ± 6.6	1677
Current smokers, *n* (%)	263 (15.7)	1678
Ex-smoker, *n* (%)	294 (20.8)	1414
Any allergy, *n* (%)	665 (39.8)	1671
Any comorbidity, *n* (%)	841 (50.4)	1668
Obesity, *n* (%)	242 (14.5%)	1668
Rhinosinusitis, *n* (%)	190 (11.4%)	1668
Rhinitis/conjunctivitis, *n* (%)	313 (18.8%)	1668
Gastroesophageal reflux disease, *n* (%)	149 (8.9%)	1668
Other comorbidities, *n* (%)	268 (16.1)	1668
Predicted FEV_1_ (or personal best PEF value), *n* (%)		
≥80%	1042 (62.3)	1673
<80%	507 (30.3)	1673
<60%	124 (7.4)	1673
Categories of asthma severity (2015 GINA), *n* (%)		
Mild	719 (42.9)	1674
Moderate	780 (46.6)	1674
Severe	175 (10.5)	1674
Level of asthma symptom control (2015 GINA)		
Well controlled	474 (28.2)	1679
^a^Partly controlled	635 (37.8)	1679
^a^Uncontrolled	570 (33.9)	1679
Current treatments, *n* (%)		
Only maintenance inhaler	492 (29.6)	1662
Only rescue inhaler	357 (21.5)	1662
Maintenance inhaler + rescue inhaler	468 (28.2)	1662
Maintenance inhaler + oral treatment	78 (4.7)	1662
Rescue inhaler + oral treatment	40 (2.4)	1662
Maintenance inhaler + rescue inhaler + oral treatment	206 (12.4)	1662
Monoclonal antibodies	23 (1.4)	1662
Type of maintenance inhaler device, *n* (%)		
Multiple-dose DPI	935 (75.2)	1244
pMDI	240 (19.3)	1244
Single-dose DPI	26 (2.1)	1244
pMDI + multiple-dose DPI	39 (3.1)	
pMDI + single-dose DPI	4 (0.3)	1244

*DPI* dry powder inhaler, *FEV*_*1*_ forced expiratory volume in 1 s, *GINA* Global Initiative for Asthma, *PEF* peak expiratory flow, *pMDI* pressurized metered-dose inhaler.

^a^Poorly controlled.

### Modifiable factors associated with asthma control

According to Global Initiative for Asthma (GINA) recommendations, 35.9% of patients had an insufficient or inadequate prescription (Table 2). In order to assess whether the prescription were adequate or not, GINA 1–5 steps were taken into account. Patients’ maintenance treatment were compared with their exacerbations in the past and their current symptoms, in order to decide whether the treatment was adequate or not, according to these 5 GINA steps. Among these patients with inadequate prescription, 82.5% had a poorly controlled (partly/uncontrolled) asthma (according to the Asthma Control Test (ACT)), whereas 56.3% of patients with adequate treatment had poorly controlled asthma (odds ratio (OR) 3.65, 95% confidence interval (CI): 2.87–4.65, *p* < 0.0001; Table 3).

**Table 2:** Prevalence of modifiable factors associated with asthma control.

Factor	*n* (%)	Number of patients evaluated
Inadequate prescription (GINA)		
Yes	604 (35.9)	1681
No	1077 (64.1)	1681
Adherence according to Morisky–Green questionnaire		
Adherent	522 (31.5)	1658
Non-adherent	1136 (68.5)	1658
Adherence according to TAI		
Adherent	381 (23.2)	1639
Non-adherent	1258 (76.8)	1639
Critical inhaler mistakes		
No errors	1394 (83.0)	1680
≥1 error	286 (17.0)	1680

*GINA* Global Initiative for Asthma, *TAI* Test of Adherence to Inhalers.

**Table 3:** Relation between asthma control by ACT criteria and modifiable factors associated with poor control.

Modified factor	Control (ACT criteria)		OR (95% CI)	*n* (%)	*p*
	Poor	Good			
Prescription					
Inadequate (A)	498 (82.5%)	106 (17.5%)	3.65 (2.87–4.65)	604 (36)	<0.0001
Adequate	606 (56.3%)	471 (43.7%)		1077 (64)	
Adherence (TAI)					
Poor (B)	866 (68.8%)	392 (31.2%)	1.80 (1.42–2.27)	1258 (74.7)	<0.0001
Good	210 (55.1%)	171 (44.9%)		381 (22.7)	
Critical mistakes					
One or more (C)	238 (83.2%)	48 (16.8%)	3.03 (2.18–4.21)	286 (17.0)	<0.0001
None	865 (62.1%)	529 (37.9%)		1394 (83.0)	
A + B	399 (85.2%)	69 (14.8%)	4.16 (3.14–5.50)	468 (27.8)	<0.0001
A + C	119 (90.1%)	13 (9.9%)	5.23 (2.92–9.36)	132 (7.8)	<0.0001
B + C	202 (84.9%)	36 (15.1%)	3.36 (2.32–4.86)	238 (14.1)	<0.0001
A + B + C	101 (93.5%)	7 (6.5%)	8.23 (3.80–17.83)	108 (6.4)	<0.0001

*ACT* Asthma Control Test, *TAI* Test of Adherence to Inhalers.

Regarding adherence to treatment, 76.8% of patient had a low adherence measured by the Test of Adherence to Inhalers (TAI) questionnaire and 68.5% by the Morisky–Green (MG) questionnaire (Table 2). Moreover, 68.8% of patients with poor adherence had poorly controlled asthma (according to the ACT), whereas 55.1% had poorly controlled asthma despite good adherence (OR 1.8, 95% CI: 1.42–2.27, *p* < 0.0001; Table 3).

As measured by the extended TAI test, almost 17% of the patients had at least one of critical error (errors in the use of the device that compromise the effectiveness of inhaled treatment) in inhaler technique (Table 2); among patients with critical mistakes, 83.2% had poorly controlled asthma (according to the ACT), versus 62.1% with poorly controlled asthma and no critical inhaler errors (OR 3.03, 95% CI: 2.18–4.21, *p* < 0.0001; Table 3). A different percentage of misuse depending on the device was a pressurized metered-dose inhaler (pMDI) or dry powder inhaler (DPI) was observed. Significantly less critical mistakes were found in Easyhaler (EH) users versus other DPI ones (Table 4), (EH: 10.3%; other DPI: 18.4%; *p* < 0.05): 10.3% of patients showed critical errors with EH, 19.5% with Accuhaler, 16.0% with Nexthaler, and 17.5% with Turbuhaler (*p* < 0.01). As a consequence (Table 4), a significant lower need to technique adjustment was observed in EH users compared to other DPI ones (*p* < 0.0001; Table 4).

**Table 4:** Number of critical mistakes and need of technique adjustment according to DPI device.

DPI device	Critical technique mistakes, *n* (%)	Need of technique adjustment, *n* (%)	Number of patients evaluated
Accuhaler	30 (19.5)	106 (68.8)	154
Easyhaler	13 (10.3)	43 (34.4)	126
Nexthaler	17 (16.0)	52 (51.5%)	106
Turbuhaler	36 (17.5)	120 (58.3%)	206

*DPI* dry powder inhaler.

### Asthma control

Regarding asthma control, 71.7% of patients had a poorly controlled asthma according to the GINA criteria and 65.7% according to ACT test. Several factors were shown to be related with poor asthma control according to the GINA criteria and the ACT test (Table 3): inadequate prescription (GINA: OR 8.05, 95% CI: 5.74–11.27; ACT: OR 3.65, 95% CI: 2.87–4.65), poor adherence to treatment (GINA: OR 1.58, 95% CI: 1.23–2.03; ACT: OR 1.8, 95% CI: 1.42–2.27), and inhaler misuse (GINA: OR 4.76, 95% CI: 3.08–7.34; ACT: OR 3.03, 95% CI: 2.18–4.21).

Each one of these risk factors (inadequate prescription, poor adherence, and inhaler misuse) has a statistically significant impact on poor asthma control (*p* < 0.0001; Table 3).

Moreover, a higher number of risk factors was related to a higher probability of having a poorly controlled asthma, reaching a maximum of 93% with the ACT and 100% with the GINA, if all three conditions were present, and 46% with the ACT and 54% with the GINA if none was present.

The control of asthma according to the GINA score compared to the ACT questionnaire score showed a moderate concordance (Kappa = 0.458; Rho = 0.709; *r*^2^ = 0.503). The corresponding tables for the GINA asthma control that are quite similar to the ACT control are shown in Supplementary Tables 1 and 2 and Supplementary Figs 1 and 2.

### Asthma knowledge questionnaire

**Fig. 1:**
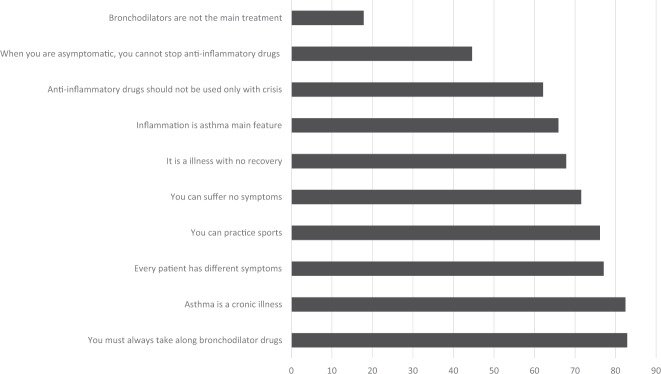
Asthma knowledge questionnaire. Percentage of correct responses to asthma knowledge questionnaire (self-administered).

**Fig. 2:**
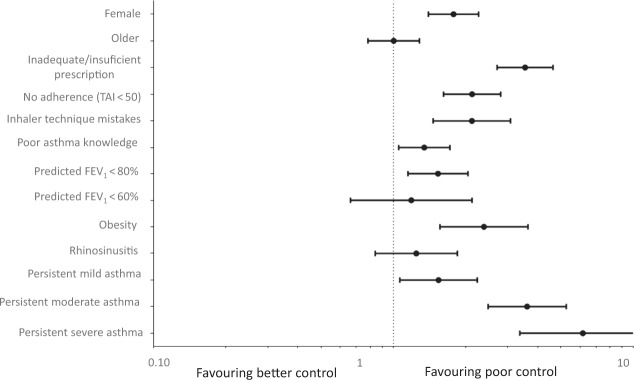
Binary logistic regression model with poor asthma control (ACT < 21) as dependent variable. This figure represents factors associated with poor asthma control.

Regarding the asthma knowledge questionnaire, the problematic questions seemed to be related to asthma treatment during both exacerbation and remission periods (Fig. 1): only 17.8% of patients knew that bronchodilators are not the main asthma treatment, and less than half of the patients knew that maintenance treatment must be continued during remission periods. Despite less than half of the patients knew the correct use of anti-inflammatory drugs, >60% of them knew that it should be used not only during crisis, because they knew that asthma is an inflammatory disease. More than 80% of patients knew that asthma is a chronic disease, and 70% of them knew that they could practice sports and could suffer no symptoms. But most of them live with a poor controlled (and consequently symptomatic) asthma (as discussed above). Despite the undoubted impact of these misconceptions on the adherence to treatment, when the impact of asthma knowledge on asthma control was evaluated, it was found to be an independent predictor of asthma poor control (Fig. 2) according to the ACT criteria.

## Discussion

Despite years of asthma guidelines implementation, in this study poor asthma control remained strongly associated with modifiable features related with therapy failure. Although many factors could be involved in poor asthma control, three factors seem to be the main causes of poor asthma control in primary care, according to previous publications^5,12^: inadequate prescription, poor adherence to treatment, and poor inhaler technique^6,9,13–15^. Obesity has a negative impact in asthma, and there are preliminary data showing the benefit of weight reduction on asthma control and quality of life^16^. However, recommendation of diet-induced weight loss in asthma patients is related with the plethora of general health benefits^17^, but it has insufficient evidence yet on asthma control^18^; for that reason, authors did not include this factor into the modifiable ones.

In our study, 71.7 and 65.7% of patients (according to the GINA and the ACT, respectively) showed a poor control of symptoms. Several studies have been conducted in primary care during the past years, with different measurable variables and design^16^. Despite their heterogeneity, all showed the same tendency toward poor asthma control. In a recent Spanish publication, Calvo et al.^19^ found poor asthma control (ACT ≤ 20) ranging from 23.4 to 75.6% in 638 asthmatic patients, depending on the patients’ medical consultation. On average, almost half of the patients showed poor control. The authors of another study^20^ have estimated that 45% of asthmatic patients who received any type of treatment in Spain had no controlled asthma (ACT ≤ 19). Asthma control in other European countries is also not encouraging: a recent Swedish study in primary care calculated that 53.6% of patients had no controlled asthma (≥600 doses of short-acting beta agonists and/or ≥1 exacerbations/year)^21^. In the European LIAISON study^22^, asthma control was assessed according to the Asthma Control Questionnaire: the percentage of patients with partly or not controlled asthma was 56.5 across 12 countries that participated in the study. In USA, the situation is similar: 50% of patients who attended primary care for a non-respiratory consultation had non-controlled asthma (ACT < 19)^23^. Most of studies show slightly lower percentage of poorly controlled patients compared with our study, which probably could be related with selection bias as the patients referred to specialized care are not expected to be asymptomatic.

More than one-third of our patients, at the time of referral from primary care, had an inadequate treatment, which was associated with a less adequate asthma control. Both providers and patients are prone to underestimate asthma severity, which can be the reason why many patients might be undertreated^9^.

The lack of time and economical resources are some of the causes of not a fully satisfactory management of asthma in the primary care setting^24^, although it is well known that a bad diagnosis and treatment suppose a detriment to the patients’ health^25^, which in turn increase care demand and health costs. In this sense, the ACT was created as a tool to detect poorly controlled asthma and, as a consequence, to identify those patients who require the most adequate treatment. It has been shown that it allows a better treatment adjustment when the physician uses it routinely^26^.

The best strategy to evaluate adherence are electronic devices^27^ that could be the gold standard^8,28^ (they are an objective method to assess patients’ adherence), but they are expensive and difficult to use for many patients. Thus self-reports seem to be the most cost-effective measure to assess it^7^, although patients are hesitant to report poor adherence in some occasions^29^. In this study, two adherence questionnaires were used, and the TAI questionnaire seems to have more sensitivity than the MG one (76 and 68.5%, respectively, have a poor adherence). Also it is shown that the TAI has a better correlation with patient real adherence^30^. Moreover, when asthma control is compared according to the ACT punctuation and the GINA criteria, a statistically significant relationship with poor adherence (ACT OR 1.8; GINA OR 1.58) is found. Similarly, in the REcognise Asthma and LInk to Symptoms and Experience (REALISE) study^11^, almost 50% of patients showed a poor adherence to treatment, and in the LIAISON study a higher rate of low adherence (according to the MG) was present in patients with poorly controlled asthma, compared to the ones with controlled asthma^22^. To stress the importance of a good adherence, it should be mentioned that its absence is linked to a higher risk of asthma exacerbations, increased use of oral corticosteroids, need of Emergency Department attendance and/or hospitalization, deterioration of forced expiratory volume in 1 s values^29,31^, and finally higher costs and poor quality of life^7^. Strategies to improve adherence have been explored, such as to review frequently the inhaler usage technique as well as patient adherence in every visit. Also electronic reminders should be used as they can be very effective^29,30,32,33^. In primary care, it has been established that an educational program could be effective in terms of improving adherence, controlling, and reducing costs. The program should involve medical practitioners as well as nursing services, after an adequate training is given^34^.

Regarding the third important factor responsible for poorly controlled asthma, inadequate inhaler technique, this study showed that 17% of the patients referred from primary care make critical mistakes, and it is directly related to bad asthma control (GINA OR 4.76; ACT OR 3.03). Serious mistakes with inhaler technique can be defined as errors potentially limiting the drug uptake and its distribution to the lungs^12^. In some studies, the percentage of critical errors is higher than the one in our study (ranging from 50 to 90%)^35^; however, without a doubt this step remains crucial in order to achieve asthma control. It has been established that a poor inhaler technique is associated with more symptoms^36^, more need for hospitalization^12^, and definitely a less effective asthma control. In a Dutch study, between 47.7 and 64.9% of patients referred to primary care presented inhaler misuse; a pragmatic intervention showed a significant improvement of this factor^37^.

The percentage of serious errors differs significantly among inhaler devices. In our study, patients showed a better performance with the DPI devices versus the MDIs, as expected. Among the DPIs, EH was associated with a lower number of critical errors compared to other devices (Turbuhaler, Nexthaler, Accuhaler). This finding is supported by previous studies that demonstrated better acceptance and satisfaction with the EH device^38,39^. In addition, several studies suggest that an explanation regarding the inhaler use and a practical demonstration of patient skills with the apparatus, as well as by taking into consideration patients’ preferences, could improve satisfaction with the inhaler and usage technique that consequently may result to a better asthma control^12,14,36,40,41^. For instance, some publications advocate for providing an explanation of inhaler technique at the Emergency Department, before patient discharge^42^.

Inadequate prescriptions, adherence to treatment, and inhaler technique overall contribute to the fact that 73.3% of patients have poorly controlled asthma (according to the GINA criteria) or 65.7% according to the ACT questionnaire. As mentioned above, these 3 parameters are essential for asthma control^5,6,9,24^, and this control becomes worse as more risk factors are present, ranging from 58.4% of poor control (ACT ≤ 20) when one condition is implicated to 93.5% when all conditions are present (or 54–100% with the GINA criteria). Our study seems to confirm such a concordance between the ACT and the GINA criteria regarding asthma control, although the GINA could identify a higher number of undercontrolled patients than the ACT.

Results regarding asthma knowledge and understanding are not encouraging, and they are remarkable factors of asthma poor control: most patients believe that bronchodilators are the main treatment in managing asthma and that the anti-inflammatory treatment can be stopped during remission periods. When asthma understanding is analyzed as predictive factor of asthma poor control through a binary logistic regression, it seems to be an independent predictor of asthma control. These concerns probably influence patients’ adherence: in the REALISE study^11^ half of the patients did not take correctly their maintenance medications, and among patients with poor control 50% did not take maintenance medication as prescribed because they thought it was not necessary. In another study^10^, almost 50% of patients believed that their asthma was well controlled, even when they had severe and persistent symptoms. Educational interventions could improve patients’ knowledge and consequently their adherence to treatment.

This study aims to determine the proportion of patients with inadequate treatment and offer an updated insight into modifiable factors involved in asthma control at primary care level. Because results are different in clinical trials and real life, as it has been widely shown^28^, we tried to use modern tools to verify whether recent advances in asthma management provided any changes in the preventable factors associated with asthma control. The result is not especially encouraging: although nowadays good recommendations for asthma control are provided, in real life this aim is far from been achieved, and an underestimation of risk appears critical between primary care physicians and their patients. Maybe educational programs with the aim to improve recognition of the importance of adherence to a proper, early, and sustained therapy and good inhaler technique for improving asthma control, as well as knowledge of asthma as chronic illness, should be taken into consideration. The mean problem about improving asthmatic patients’ education by physicians and nurses is probably the lack of time addressed to this aim, especially in some Health Care Systems. This is a common problem in chronic diseases. Probably, the first step could be the obligatory establishment of a time addressed only to education to the knowledge of asthma disease and the correct use of the devices for its treatment. These kinds of interventions could improve outcomes and cost-effectiveness as it was demonstrated by the Finnish program^34^.

There are several limitations in this study. No reason for patients’ referral from primary care to pneumologist or allergologist was recorded, which could create a bias during patients’ selection. Furthermore, in primary care all tools of GINA treatment steps are not available, so patients with severe asthma hardly would be well controlled at primary care. Patients were enrolled during a relatively short period of time (from September to December): patients with seasonal respiratory symptoms, due to a specific allergy profile, were not enrolled in the study. In this study, the ACT cut-off point was established at 21, while it was fixed at 19–20 in other studies. An overestimation of asthma poor control could be present in this study. Regarding critical mistakes, assessing inhaler technique may be challenging because some aspects of the technique are subjective, such as “synchronization between actuation and inhalation” when evaluating pMDI devices or “inhale deeply” when evaluating DPI devices. However, the investigators who assessed inhaler technique were well trained and had lots of experience educating patients; therefore, we think that the reliability of inhaler technique evaluation is good. In addition, the use of pMDI with spacer devices was not evaluated. However, the use of a spacer is generally recommended for children and older people, and its use is not common in adults^43^. Finally, study population is heterogeneous regarding comorbidity, previous diagnostic and treatment, and habits. Although this heterogeneity lies closer to real life, it is s not easy to extrapolate these results to a concrete population. Anyway, the aim of the current study is not to criticize primary care management of asthmatic patients but to get a picture of referred asthmatic patients’ condition.

Our results suggest that poor asthma management, adherence to treatment, inhaler technique, and poor asthma knowledge constitute, at primary care level, critical factors resulting in reduced asthma control. Many patients do not receive adequate treatment and adherence to therapy is poor when referred to a Specialist. In addition, patients showing critical mistakes in the inhaler technique is a frequent finding.

This study demonstrates that there is yet room for improvement by acting on these modifiable factors that appear as key opportunities for the improvement of asthma management at primary care level by optimizing therapy, retraining on inhaler technique and asthma knowledge, and developing new tools to improve adherence to treatment.

## Methods

### Study design and participants

A cross-sectional multicenter observational study was conducted in patients aged ≥18 years who made use of any type of inhaler device and were referred from primary care to a pneumologist or allergologist for the first time for every kind of reason. Data were collected from September to December 2016. Exclusion criteria were the lack of a signed informed consent and incapacitating illness or mental disease, making the participation to the study, according to the research criteria, difficult. All patients were undergone to a spirometry and a bronchodilator test in order to confirm/exclude asthma diagnose.

### Study measurements

The following main variables were assessed: appropriate asthma treatment according to the GINA recommendations^44^ (GINA treatment steps 1–5 were used to assess the appropriateness of current patients’ treatment, according to asthma control at the visit time. For this evaluation, GINA criteria of asthma control were used, as explained below), treatment adherence according to the MG questionnaire (patients were classified into reliable or not reliable according to a punctuation from 0 to 4, in which 0 = reliable and ≥1 = not reliable), adherence to inhalers (TAI ≥ 50 reliable; <50 not reliable)^45,46^, and critical errors with patient inhaler technique^46^ identified through item 12 of the TAI questionnaire. Only the main inhaler for maintenance therapy was evaluated. This item consists of a practical demonstration of patients’ inhaler technique, in addition to discover critical errors by the physician. This item shows 2 categories of patients: with ≥1 or without critical errors. Additional patient knowledge about asthma was evaluated through a questionnaire from the GEMA educational material^47^ (Supplementary Table 3).

The following clinical data were collected: asthma severity according to the 2015 GINA criteria^44^, comorbidities (allergy, occupational exposure to allergens or irritants, smoking, etc.), and asthma control according to both GINA and ACT criteria^44,48^. According to GINA, these items were taken into account: patients’ day/nighttime symptoms (<2 times/week daytime symptom and no nighttime symptoms), treatment to relieve symptoms (<2 times/week), and no activity limitations in the past 4 weeks. Thus patients were classified into 2 categories: well controlled (with all previous items), partially/not controlled (with 1–4 of previous items). According to the ACT, patients were classified into the same 2 categories, but in this case established through 5 items of the ACT validated Spanish version (≥21: well controlled, <21: partially/not controlled). Patients having a body mass index >30 were diagnosed as obese.

All data were collected in a single visit, through an online patient notebook, proportioned to the needs of the study. Ethics Committee permission (Hospital Clinic de Barcelona, Registration number HCB/2016/0647) was obtained, and the study was performed according to the Helsinki Declaration (1964). Informed consent was obtained from all participants.

### Statistical analysis

Statistical inference was analyzed with the Pearson’s chi-squared test (*χ*^2^) for categorical data and with analysis of variance test for continuous data. A binary logistic regression was used in order to establish the independence of detected factors (OR). Relationship and concordance between ACT/GINA about asthma control were expressed through Cohen’s *k* coefficient, Spearman’s correlation coefficient, and *R* square (*R*^2^). All data were analyzed with SPSS 20.0 version.

### Reporting summary

Further information on research design is available in the Nature Research Reporting Summary linked to this article.

## Supplementary information

Supplementary Information

Reporting Summary

## Data Availability

The data that support the findings of this study are available from CLEVER RESEARCH but restrictions apply to the availability of these data, which were used under license for the current study, and so are not publicly available. Data are, however, available from the authors upon reasonable request and with permission of ORION PHARMA.
