# Supplementary material for: Prevalence of modifiable factors limiting treatment efficacy of poorly controlled asthma patients: EFIMERA observational study
Source: NPJ Prim Care Respir Med. 2020 Jul 31;30:33. doi: 10.1038/s41533-020-00189-6 (PMC7395116; doi:10.1038/s41533-020-00189-6)
Supplement: Supplementary file 1 — Supplementary Information [file 41533_2020_189_MOESM1_ESM.docx]

**Supplementary Table 1. Relation between asthma control by GINA criteria and three different modifiable factors associated with poor control.**

|  | | **Adequate prescription** | | **Inadequate prescription** | | **Total** | | **OR (95%CI)** | **p** |
| --- | --- | --- | --- | --- | --- | --- | --- | --- | --- |
|  | | n | % | n | % | n | % |  |  |
| Asthma control | Control | 405 | 37.7 | 42 | 7.0 | 447 | 26.7 | 8.05 (5.74-11.27) | <0,0001 |
|  | Poor control | 670 | 62.3 | 559 | 93.0 | 1229 | 73.3 |  |  |
|  | Total | 1075 | 100.0 | 601 | 100 | 1676 | 100.0 |  |  |

|  | | **Good adherence (TAI)** | | **Poor adherence (TAI)** | | **Total** | | **OR (95%CI)** | **p** |
| --- | --- | --- | --- | --- | --- | --- | --- | --- | --- |
|  | | n | % | n | % | n | % |  |  |
| Asthma control (GINA) | Control | 128 | 33.8 | 306 | 24.4 | 434 | 26.5 | 1.58 (1.23-2.03) | <0,0001 |
|  | Poor control | 251 | 66.2 | 950 | 75.6 | 1201 | 73.5 |  |  |
|  | Total | 379 | 100.0 | 1256 | 100 | 1635 | 100.0 |  |  |

|  | | **No errors** | | **≥1 error** | | **Total** | | **OR (95%CI)** | **p** |
| --- | --- | --- | --- | --- | --- | --- | --- | --- | --- |
|  | | n | % | n | % | n | % |  |  |
| Asthma control (GINA) | Control | 423 | 30.4 | 24 | 8.4 | 447 | 26.7 | 4.76 (3.08-7.34) | <0,0001 |
|  | Poor control | 967 | 69.6 | 261 | 91.6 | 1228 | 73.3 |  |  |
|  | Total | 1390 | 100.0 | 285 | 100 | 1675 | 100.0 |  |  |

GINA: Global Initiative for Asthma. TAI: Test of Adherence to Inhalers.

**Supplementary Table 2. Relation between asthma control by GINA criteria and modifiable factors associated with poor control and these factors between them.**

| **Modified factor** | | **Control**  **(GINA criteria)** | | **OR (95%CI)** | **n** | **p** |
| --- | --- | --- | --- | --- | --- | --- |
|  | | Poor | Good |  | | |
| Prescription | Inadequate (A) | 559 (93.0%) | 42 (7.0%) | 8.05 (5.74-11.27) | 601 | <0.0001 |
|  |  |  |  |  |  |  |
| Adherence (TAI) | Poor (B) | 950 (75.6%) | 306 (24.4%) | 1.58 (1.23-2.03) | 1256 | <0.0001 |
|  |  |  |  |  |  |  |
| Critical mistakes | One or more (C) | 261 (91.6%) | 24 (8.4%) | 4.76 (3.08-7.34) | 285 | <0.0001 |
|  |  |  |  |  |  |  |
| A+B | | 440 (35.8%) | 27 (6.0%) | 8.66 (5.77-13.0) | 1677 | <0.0001 |
| A+C | | 130 (10.6%) | 1 (0.8%) | 52.7 (7.34-378.0) | 1677 | <0.0001 |
| B+C | | 220 (17.9%) | 18 (4.0%) | 5.19 (3.16-8.5) | 1677 | <0.0001 |
| A+B+C | | 108 (100.0%) | 0 (0.0%) |  | 1673 | <0.0001 |

GINA: Global Initiative for Asthma. TAI: Test of Adherence to Inhalers.

**Supplementary Figure 1. Schematic representation of the relationship between three modifiable factors associated with poor control (data details in Supplementary Table 2).**


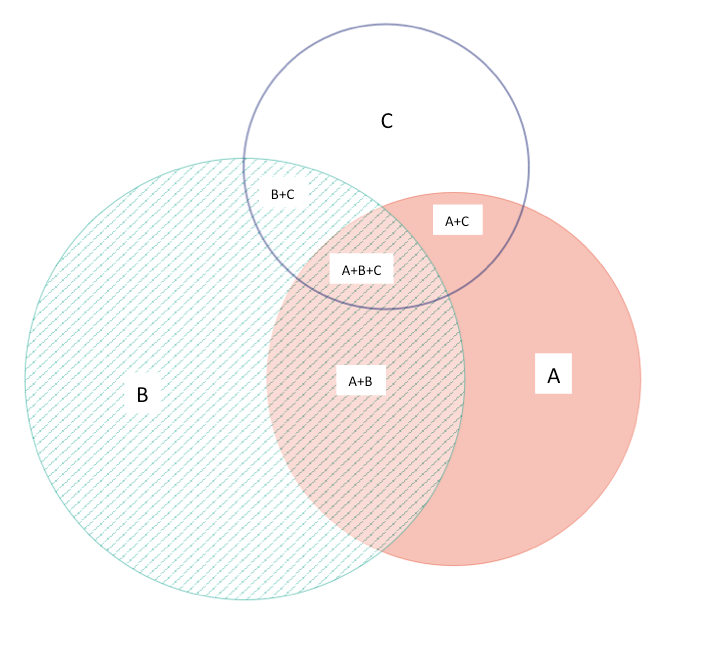


A: Inadequate prescription

B: Poor adherence

C One or more critical mistakes

**Supplementary Figure 2. Binary logistic regression model with poor asthma control by GINA as dependent variable.**


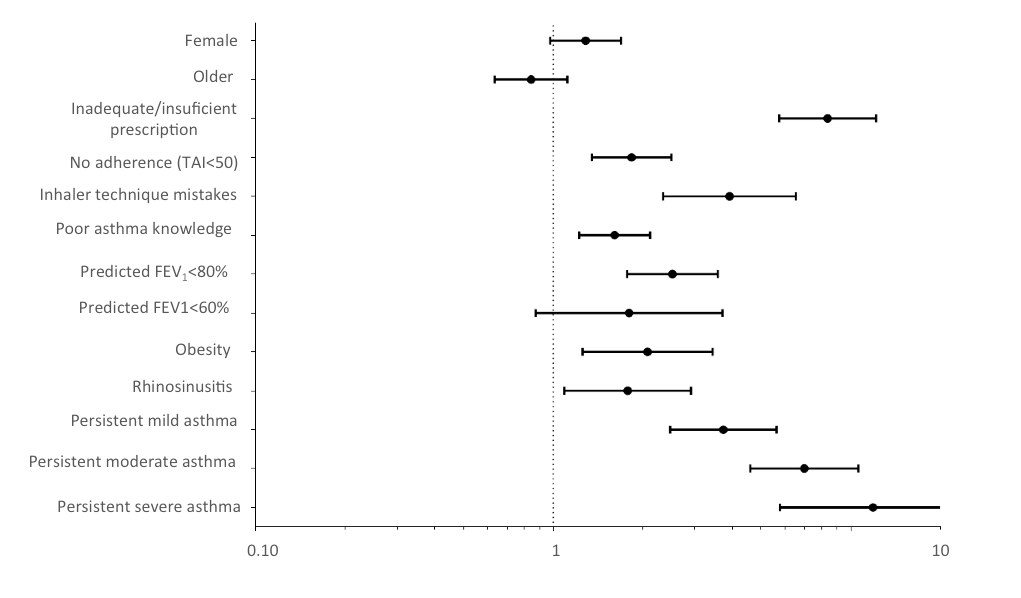


Forced Expiratory Volume in one second. GINA: Global Initiative for Asthma. TAI: Test of Adherence to Inhalers.

**Supplementary Table 3. ASTHMA KNOWLEDGE QUESTIONNAIRE (Self-administered)**

This questionnaire has been developed based on the self-administered asthma knowledge questionnaire included in the 2009 GEMA Educators Guide. Available from: <http://www.seicap.es/gema-educadores_30428.pdf>.

| Is asthma a chronic disease? | Yes | No |
| --- | --- | --- |
| Is asthma a curable disease? | Yes | No |
| Is inflammation the main characteristic of asthma? | Yes | No |
| Can you do sports? | Yes | No |
| Are bronchodilators the main treatment? | Yes | No |
| Do all patients have the same symptoms? | Yes | No |
| Are anti-inflammatories used only to treat exacerbations? | Yes | No |
| Can you remain stable with hardly any symptoms? | Yes | No |
| When you are well, can you stop the anti-inflammatory medication? | Yes | No |
| Should you always carry bronchodilator medication? | Yes | No |
