# Supplementary material for: Prevalence of modifiable factors limiting treatment efficacy of poorly controlled asthma patients: EFIMERA observational study
Source: NPJ Prim Care Respir Med. 2020 Jul 31;30:33. doi: 10.1038/s41533-020-00189-6 (PMC7395116; doi:10.1038/s41533-020-00189-6)
Supplement: Supplementary file 2 — Reporting Summary [file 41533_2020_189_MOESM2_ESM.pdf]

nature research

Corresponding author(s): DBPR- number-NPJPCRM-00837

Last updated by author(s): Jun 25, 2020

Reporting Summary

Nature Research wishes to improve the reproducibility of the work that we publish. This form provides structure for consistency and transparency in reporting. For further information on Nature Research policies, see Authors & Referees and the Editorial Policy Checklist .

Statistics

For all statistical analyses, confirm that the following items are present in the figure legend, table legend, main text, or Methods section.

n/a	Confirmed	
☐	☒	The exact sample size (*n*) for each experimental group/condition, given as a discrete number and unit of measurement
☒	☐	A statement on whether measurements were taken from distinct samples or whether the same sample was measured repeatedly
☒	☐	The statistical test(s) used AND whether they are one- or two-sided<br>*Only common tests should be described solely by name; describe more complex techniques in the Methods section.*
☐	☒	A description of all covariates tested
☐	☒	A description of any assumptions or corrections, such as tests of normality and adjustment for multiple comparisons
☐	☒	A full description of the statistical parameters including central tendency (e.g. means) or other basic estimates (e.g. regression coefficient) AND variation (e.g. standard deviation) or associated estimates of uncertainty (e.g. confidence intervals)
☐	☒	For null hypothesis testing, the test statistic (e.g. *F*, *t*, *r*) with confidence intervals, effect sizes, degrees of freedom and *P* value noted<br>*Give P values as exact values whenever suitable.*
☒	☐	For Bayesian analysis, information on the choice of priors and Markov chain Monte Carlo settings
☒	☐	For hierarchical and complex designs, identification of the appropriate level for tests and full reporting of outcomes
☒	☐	Estimates of effect sizes (e.g. Cohen's *d*, Pearson's *r*), indicating how they were calculated

Our web collection on statistics for biologists contains articles on many of the points above.

Software and code

Policy information about availability of computer code

Data collection	Online patient notebook, proportioned to the needs of the study
Data analysis	SPSS v 20.0

For manuscripts utilizing custom algorithms or software that are central to the research but not yet described in published literature, software must be made available to editors/reviewers. We strongly encourage code deposition in a community repository (e.g. GitHub). See the Nature Research guidelines for submitting code & software for further information.

Data

Policy information about availability of data

All manuscripts must include a data availability statement. This statement should provide the following information, where applicable:

- Accession codes, unique identifiers, or web links for publicly available datasets
- A list of figures that have associated raw data
- A description of any restrictions on data availability

The data that support the findings of this study are available from CLEVER RESEARCH but restrictions apply to the availability of these data, which were used under license for the current study, and so are not publicly available. Data are however available from the authors upon reasonable request and with permission of ORION PHARMA.

Field-specific reporting

Please select the one below that is the best fit for your research. If you are not sure, read the appropriate sections before making your selection.

Life sciences study design

All studies must disclose on these points even when the disclosure is negative.

Sample size	The calculation of the simple size has been made with the intention to determine the prevalence of factors that diminish asthma´s treatment ´s efficacy .Given that the expected proportion of the presence of each risk factor, a formula for the estimation of a proportion in statistically infinite populations was used to calculate sample size N= (Z2 x p x q) /d2
Data exclusions	No data were excluded
Replication	Not apply
Randomization	Not apply
Blinding	Not apply

Reporting for specific materials, systems and methods

We require information from authors about some types of materials, experimental systems and methods used in many studies. Here, indicate whether each material, system or method listed is relevant to your study. If you are not sure if a list item applies to your research, read the appropriate section before selecting a response.

Materials & experimental systems

n/a	Involved in the study	
☒	☐	Antibodies
☒	☐	Eukaryotic cell lines
☒	☐	Palaeontology
☒	☐	Animals and other organisms
☐	☒	Human research participants
☐	☒	Clinical data

Methods

n/a	Involved in the study	
☒	☐	ChIP-seq
☒	☐	Flow cytometry
☒	☐	MRI-based neuroimaging

Human research participants

Policy information about studies involving human research participants

Population characteristics	Population characteristics: male and femal >18 years old, with a diagnostic of asthma in Primary care, and in treatment with inhaler devices (short acting beta2 agonists, corticosetroids +/- long acting beta2 agonists)
Recruitment	Consecutive patients fulfilling inclusion criteria
Ethics oversight	Ethics Committee permission (Registration 129 number. HCB/2016/0647)

Note that full information on the approval of the study protocol must also be provided in the manuscript.

Clinical data

Policy information about clinical studies

All manuscripts should comply with the ICMJEguidelines for publication of clinical research and a completedCONSORT checklist must be included with all submissions.

Clinical trial registration	No trial registration
Study protocol	Full protocol could be accessed by contacting with corresponding author
Data collection	From 1st september to 31 december 2016 in more than 200 specialized ambulatory care centers around Spain
Outcomes	outcome1: determine the appropriateness of prescribed treatment as well as the presence of poor adherence and critical mistakes in the inhalation technic, in patients referred from primary care (Pearson's chi-squared test (χ2) for categorical data, ANOVA test for continuous data)....... outcome 2: to study the relationship between these factors and poor asthma control (binary logistic regression).......
